# Gluteal Necrosis and Lumbosacral Plexopathy in a Diabetic Patient after Renal Transplantation

**DOI:** 10.1155/2015/976912

**Published:** 2015-12-17

**Authors:** M. A. Asgari, N. Masoumi, H. Argani

**Affiliations:** ^1^Urology Department, Shahid Modarres Hospital, Shahid Beheshti Medical University, Saadat Abad, Tehran 1998734383, Iran; ^2^Nephrology Department, Shahid Modarres Hospital, Shahid Beheshti Medical University, Saadat Abad, Tehran 1998734383, Iran

## Abstract

A 34-year-old diabetic patient underwent a renal transplant which was complicated by right side lower extremity paresis and numbness with gluteal necrosis. The main reason was ligation of internal iliac artery of the same side as a result of extensive microvascular obstruction due to severe atheromatous plaque. This is a rare complication which is mostly reported in aneurysmal patients after bypass surgery. The gluteal necrosis is a serious complication which, as in our patient, resulted in patient's death in most of the reported cases. Because of catastrophic nature of this condition, identifying preventive measures is extremely important.

## 1. Case Presentation

A 34-year-old white male with the background of type 1 diabetes which was complicated by retinopathy and nephropathy (under hemodialysis since July 2014) scheduled for kidney transplantation and the surgery was done at April 2015 from a live donor (sibling). Opium addiction and hypertension from 2 years ago were other main points in his past medical history. In preoperative imaging (CT) the significant finding was heavily calcified mural atherosclerotic plaques in abdominal and iliac arteries.

During surgery, the right internal iliac artery had severe atheromatous plaque which after endarterectomy was anastomosed to renal artery in an end-to-end fashion, with ligation of anterior and posterior divisions. Because of insufficient renal filling and no change in renal color, anastomosis was revised in the same location with acceptable outcome in color and firmness.

In recovery room he had an apnea with drop in oxygen saturation, which required resuscitation. He was then transferred to ICU and was under observation for the next 24 hours. After becoming stable, he was transferred to ward with no complication, although the urine output was <500 cc/day the whole time. Doppler showed normal flow in the transplanted kidney.

**Figure 1:**
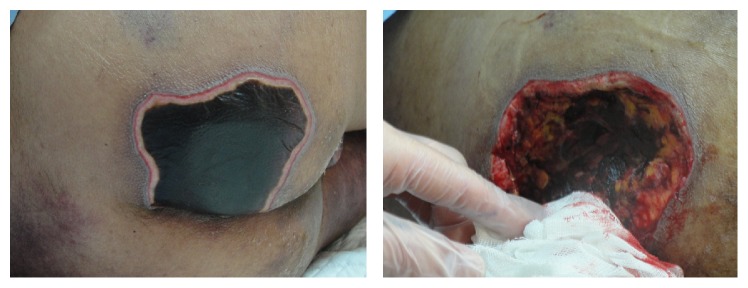
Necrotic site in the right hip which progressed to a large ulcer in the following days.

On postoperative day 2, he complained of pain and inability to move his right leg since the night before. On physical exam, there was an ischemic area 2 × 2 cm in diameter on the right hip. This ischemic area progressed to a large necrotic area 7 × 7 cm in the following days (Figure 1). Neurologic exam revealed diminished strength in hip flexion (3/5) and knee extension (3/5) with 4/5 strength in ankle dorsiflexion and plantar flexion and toe extension. Sensation was poor in all the right extremity and the patellar and ankle reflexes were lost. He also had no urine and fecal control. EMG study documented profound sensorimotor polyneuropathy and right lumbosacral plexopathy. Lower limb Doppler US was unremarkable.

**Figure 2:**
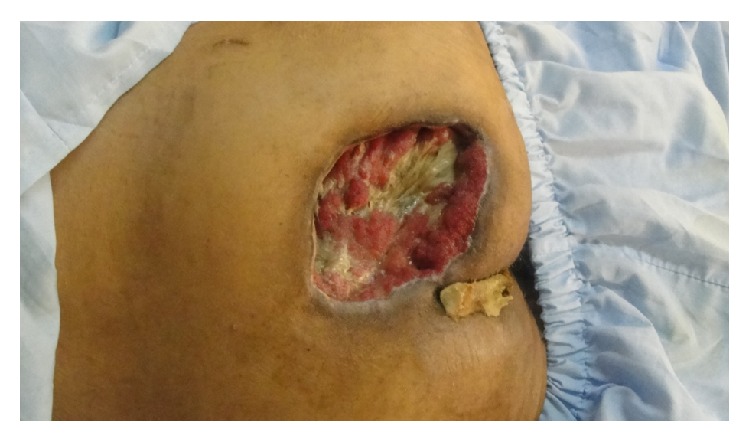
Improvement is ulcer area 2 months later.

On the following days, because of expanding tissue necrosis, he underwent serial wound debridement. Meanwhile, the transplanted kidney did not function (humeral rejection) and was ultimately removed. In the following weeks, mild improvement was seen in motor control and continence and also the wound showed promising signs of granulation tissue (Figure 2).

Unfortunately the patient died three months postoperatively of sepsis induced multiple organ failure.

## 2. Discussion

Acute lumbosacral plexopathy and gluteal necrosis are a rare phenomenon in renal transplant patients. The first report of plexopathy dates back to 1990 by Hefty et al. [1], which occurred in four diabetic patients after transplantation. In another report by Jablecki et al. [2], paraplegia following renal transplantation is seen after ligation of hypogastric artery. Similar finding of lumbosacral plexopathy was mentioned after dual kidney transplantation [3] and even as a presentation of iliac artery pseudoaneurysm in transplanted patients [4]. There are also other reports of femoral neuropathy because of compression of malpositioned retractors during surgery [5].

Due to vast arterial network and collaterals in pelvis, ligation of internal iliac artery usually has no adverse outcome. This network stems from anterior and posterior division of bilateral internal iliac artery which supplies blood to sciatic nerve and gluteal musculature. In detail, inferior gluteal artery is a branch of anterior division which supplies the pelvic viscera, the lower hip, and the back of the thigh. The posterior division gives rise to superior gluteal artery, which supplies the gluteal musculature, the femoral nerve, and the sciatic nerve roots [6].

Atheromatous plaques are always a concern in old and especially diabetic patients and in renal failure condition this process accelerates to an even total obstruction. Internal iliac artery is a favorite artery to use in transplantation because of flexibility in length and also because of drawbacks of external iliac artery use like ischemia of lower extremity [1]. Meanwhile, in diabetic patients, an end-to-end anastomosis of ipsilateral artery, while there is an extensive vascular and microvascular involvement, ends up in ischemic complications like our patient.

There are other examples of similar events in aneurysmal patients following bypass surgery [7–11]. Iliopoulos et al. reported eleven patients of aortic aneurysm with hypogastric artery ligation. In eight of them with bilateral hypogastric ischemia, all experienced neurologic deficit. This was accompanied by gluteal necrosis in four, rectal ischemia in two, and anal and bladder sphincter dysfunction in three [7]. Hundred percent of patients with gluteal necrosis passed away in the following weeks; however, mortality rate in patients without necrosis was 25%. This mortality rate was also reported in Picone et al.'s series as well [9].

In a nutshell, our patient's scenario of lower extremity paresis and numbness with gluteal necrosis and EMG findings is best explained by infarction in the area supplied by right superior gluteal artery and its branches to the gluteal musculature, femoral nerve, and sciatic nerve roots.

## 3. Conclusion

Given the catastrophic nature of this complication and high mortality rate, identifying means to prevent such events is essential. Especially in diabetic patients with extensive atheromatous disease, thorough physical exam of peripheral pulses and angiography to determine the patency of pelvic vasculature are prudent. This may allow preplanning the transplant site and avoiding the ischemic complications. One option in these sorts of patients is orthotopic kidney transplantation with use of the splenic artery and native left renal vein for vascular reconstruction.
